# Pediatric Falls: Are Monkey Bars Bad News?

**DOI:** 10.7759/cureus.3548

**Published:** 2018-11-05

**Authors:** David Migneault, Albert Chang, Edward Choi, Quynh Doan

**Affiliations:** 1 Emergency Medicine, University of British Columbia, Vancouver, CAN; 2 Emergency Medicine, British Columbia Children's Hospital, Vancouver, CAN

**Keywords:** fall, pediatric injury, fracture, monkey bars, playground

## Abstract

Background

Falls are a leading cause of childhood trauma and are the most common mechanism of injury seen in the emergency department (ED). Playground injuries represent a significant fraction of these falls.

Objectives

This study aims to compare the frequencies of fractures from monkey bars to other types of falls and to explore the statistical associations between the types of injuries.

Methods

We conducted a cross-sectional study through a retrospective chart review of all British Columbia Children’s Hospital ED visits between March 2011 and February 2012. We manually extracted data from ED visits for falls in children two to 17 years of age and used descriptive statistics to report the frequencies of injuries and outcomes. We conducted multivariate logistic regression analyses to compare the odds of fractures associated with various types of falls.

Results

We reviewed 43,579 ED visits, of which 3,184 (7.3%) were falls. The most common types were from a standing height (42.5%), falls at home (16.2%), and at the playground (14.3%). Peaking in school-age children, these falls resulted in a diagnosis of fracture (37.3%), soft tissue contusion (20.1%), laceration/abrasion (19.4%), and minor head injury (15.8%). We identified 151 falls from monkey bars, among which 64.2% resulted in a fracture. The odds of a fracture following a fall from monkey bars was 3.1 times that of falls from all other causes.

Conclusions

ED physicians should have a higher suspicion for a diagnosis of fracture if a child reportedly fell from monkey bars. It is warranted to educate parents and educators on the risks associated with the play on these climbing structures.

## Introduction

Falls account for 25%-52% of all treated child injuries. They are a leading cause of nonfatal childhood trauma and are the most common mechanism of injury seen in the emergency department (ED) [1-3]. While mortality due to a fall is rare among children, the rates of hospitalization and ED visits are high [3]. Annually, in North America, approximately three million children who fall visit the ED and the resulting injuries represent the second leading cause of pediatric hospitalization [2,4-5].

Mathison and Agrawal suggest that the interplay of three major epidemiological factors contributes to fall-related pediatric injuries: demographics (age, sex, race, ethnicity, socioeconomic status, season, and obesity), behavior (risk-taking), and bone health (bone density, eating disorders, nutrition, prescription drugs, performance-enhancing drugs, smoking, and genetics) [6]. A detailed analysis of the American National Electronic Injury Surveillance System (NEISS) revealed that 83.9% of ED visits secondary to playground equipment injuries were related to monkey bars, swings, and slides [7]. A British study showed that the risk of injury or fracture due to a fall from monkey bars was twice the risk for all climbing-frames and seven times greater than the risk for swings or slides [8]. We hypothesize that a child presenting to the ED of a children’s hospital in Vancouver (Canada) after a fall from monkey bars has a significant likelihood of having a fracture.

This study aims to compare the frequency of fractures from monkey bars to fractures from other types of falls and to explore the relationships between the types of injuries and the causes of falls. It describes the outcomes from common causes of falls requiring ED assessments and may guide the development of preventive measures in a Canadian environment.

## Materials and methods

The methodological standards described by Gilbert et al. and Worster et al. were used to design this retrospective cross-sectional chart review at the British Columbia Children’s Hospital (BCCH) Pediatric ED, a tertiary care trauma center with over 40,000 annual visits [9-10]. All ED visits from March 2011 to February 2012 were manually reviewed. All charts of children aged 24 months to 17 years, who presented to the ED with a “fall” as the chief complaint or as a mechanism of injury in the clinical history, were included for data extraction. Subsequent visits for the same injury and falls suffered by children with central nervous system disorders, global developmental delay, neuromuscular disorders, osteogenesis imperfecta, or those who were assaulted or pushed were excluded. To account for seasonal variation and provide a comprehensive description of our population, a year’s worth of ED visits were reviewed to meet our sample size.

The primary investigator trained two research assistants to use a standardized electronic data extraction form. Either the primary investigator and a research assistant or two research assistants reviewed charts for inclusion from the months of April, July, and October. If a disagreement was noted, the third reviewer would decide if the case was to be included or excluded. An inter-extractor agreement for inclusion and exclusion was assessed by Cohen’s Kappa statistic. The variables collected were the Canadian Triage and Acuity Scale (CTAS) score, age, sex, type of injury, anatomical location of the injury, imaging modality required, need for fracture reduction, primary final diagnosis, patient’s discharge plan, and month and time of the ED visit.

We analyzed the data using descriptive statistics and performed a multivariate logistic regression analysis for falls from monkey bars adjusted for age, sex, season, anatomical location, and a diagnosis of fracture. A univariate analysis was conducted using the same variables for a fracture outcome secondary to fall from monkey bars. Only those variables with a clinical statistical significance were included in the multivariable analyses (Odds Ratio (OR) not 1.0). The sample size required to estimate the proportion of fractures among subjects with a fall from monkey bars using a 95% CI level and 5% precision was calculated as 369 subjects, assuming at least 59% would have a fracture as per Waltzman et al. [11].

This study was reviewed and approved by the Research Ethics Board at the University of British Columbia.

## Results

We manually reviewed 43,579 ED visits. Of these, 3,184 (7.3%) children met our inclusion criteria for a fall. Based on Blackman and Koval, the inter-reviewer agreement on visits to include in our review of falls was almost perfect, with the lowest Kappa value comparing an agreement between one of three possible pairs of reviewers being 0.9 [12]. The study subjects’ demographic characteristics among all patients with falls and among those with an associated fracture are presented in Table 1. The type of fall is also reported.

**Table 1:** Study Population Demographic and Types of Falls Distribution

	ALL FALLS			FALLS WITH FRACTURES			
Total N: 3184	N (3184)	%	95% CI		N (1189)	%	95% CI
AGE							
2 to 3	729	22.9	21.4 - 24.4		180	15.1	13.1 - 14.1
4 to 5	585	18.4	17.1 - 19.8		196	16.5	14.4 - 18.6
6 to 7	441	13.9	12.7 - 15.1		201	16.9	14.8 - 19.0
8 to 9	362	11.4	10.3 - 12.5		161	13.5	11.6 - 15.4
10 to 11	411	12.9	11.7 - 14.1		188	15.8	13.7 - 17.9
12 and up	651	20.4	19.1 – 21.9		261	22.0	19.7 - 24.4
Not documented	5	0.2	0.1 – 0.4		2	0.2	0.0 - 0.45
SEX							
Male	1799	56.5	54.8 – 58.2		663	55.8	53.0 - 58.6
Female	1378	43.4	41.6 – 45.0		525	44.2	41.4 - 47.0
Not documented	7	0.2	0.1 – 0.5		1	0.1	0.0 - 0.28
TYPE OF FALL							
From standing height	1353	42.5	40.8 - 44.2		467	39.3	36.5 - 42.1
At Home^*^	516	16.2	14.9 - 17.5		132	11.1	9.3 - 12.9
Playground	454	14.3	13.1 - 15.5		240	20.2	17.9 - 22.5
Sports	405	12.7	11.5 - 13.9		166	14.0	12.0 - 16.0
On wheels (non-motorised)	357	11.2	10.1 - 12.3		152	12.8	10.9 - 12.7
Other^**^	99	3.1	2.5 - 3.7		32	2.7	1.8 - 3.6

**Figure 1:**
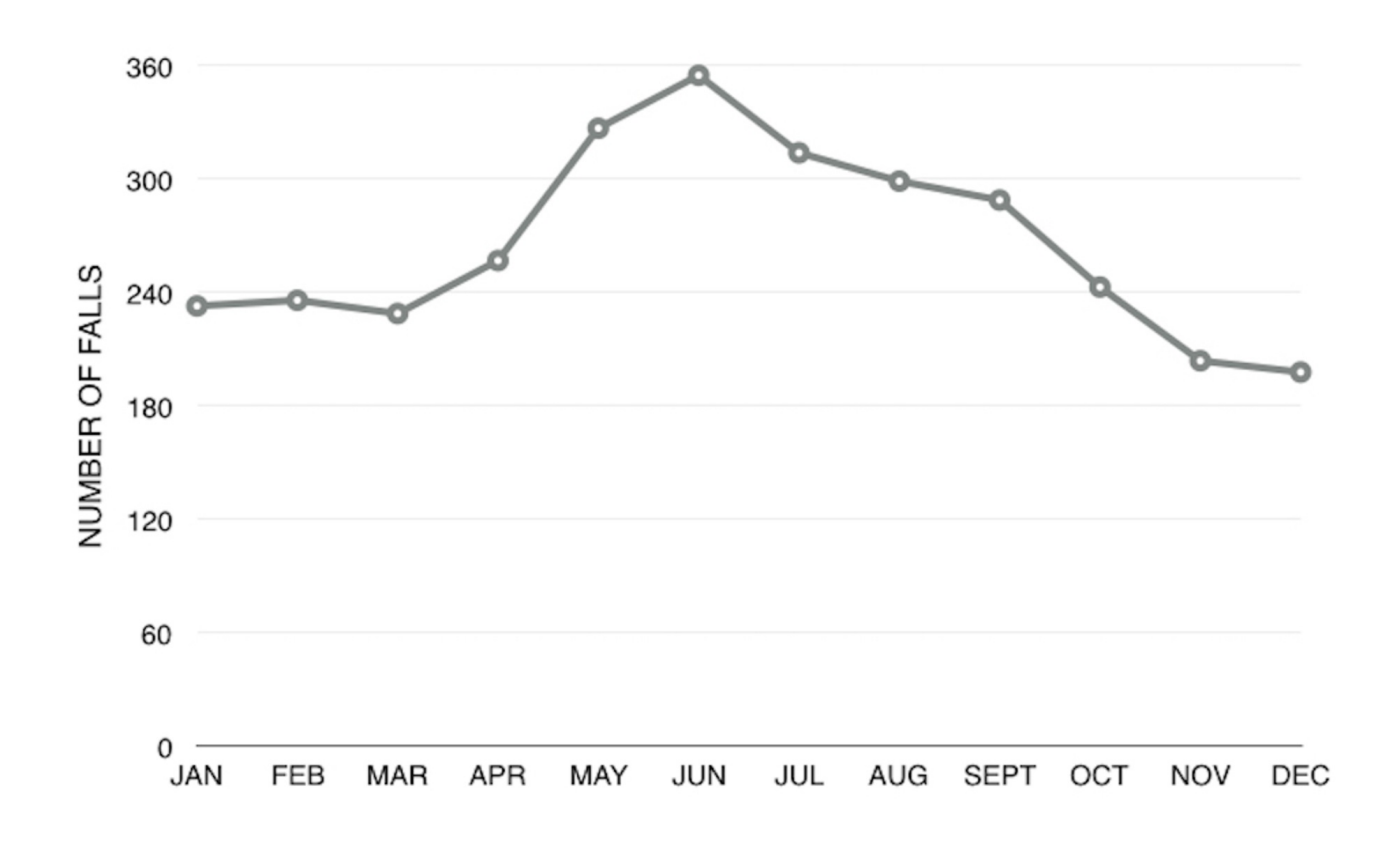
Linear Chart of Number of Falls per Month

Most falls occurred in the summer months, with 31.3% between May and July (Figure 1).

**Figure 2:**
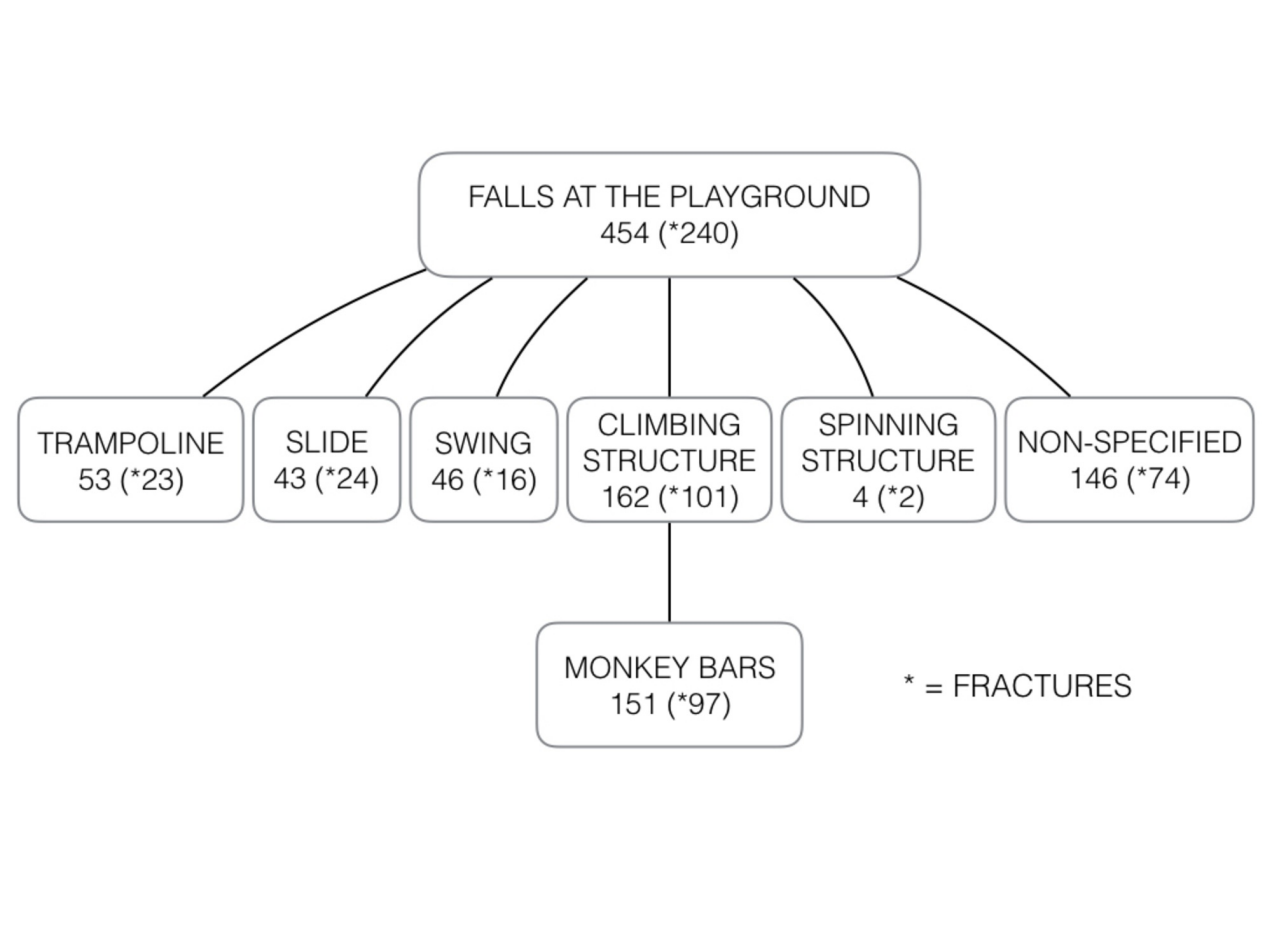
Flow Diagram of Falls at the Playground by Mechanisms and Diagnosis

The largest proportion of subjects reviewed fell from a standing height (42.5%), many while running. Falling from a standing height was also the most frequent type of fall among subjects with fractures (39.3%), with a third of falls from a standing height resulting in a fracture (34.5%). Lacerations/abrasions were present in 23.1% of children who fell from a standing height followed by non-specific soft tissue contusions or sprains (19.8%). The second most frequent type of fall were falls from pieces of home furniture or from parents’ arms, accounting for 16.2% of all falls. Out of all falls from furniture/arms, 25.6% led to a fracture, which represents 11.1% of all fractures. Slightly less frequent falls from playground equipment, non-motorized wheels, and during organized sports activities resulted in higher proportions of fractures (52.9%, 42.6%, and 41.0% respectively) (Figure 2).

A total of 151 falls (4.7%) were specifically documented to be from monkey bars and 97 (64.2%) of these children suffered a fracture (Table 2, Table 3). The majority of ED visits for falls from monkey bars were in children aged four to eight years old (78.8%) and 70.6% of them were diagnosed with a fracture. Adjusted for age and sex, the odds of having a fracture from a fall from monkey bars was 3.1 (95% CI 2.2-4.5) times higher than to those falling from all other causes. The univariate analysis between fractures and season and between fractures and anatomical location revealed statistically insignificant results.

**Table 2:** Frequency of Falls and Fractures from Monkey Bars by Age and Sex

	MONKEY BARS			MONKEY BARS FALLS WITH FRACTURES		
	N (151)	%	95% CI	N (97)	%	95% CI
AGE						
2 to 3	13	8.6	4.1 - 13.1	3	2.0	0.0 - 4.8
4 to 5	50	33.1	25.6 - 40.6	31	32.0	22.7 - 41.3
6 to 7	69	45.7	37.8 - 53.7	53	54.6	44.7 - 64.5
8 to 9	15	9.9	5.1 - 14.7	9	9.3	3.5 - 15.1
10 to 11	4	2.7	0.1 - 5.3	1	1.0	0.0 - 3.0
12 and up	0	0	-	0	0	-
SEX						
Male	71	47.0	39.0 - 55.0	42	43.3	33.4 - 53.2
Female	80	53.0	45.0 - 61.0	55	56.7	46.8 - 66.6

**Table 3:** Diagnosis and Anatomical Location of Falls from Monkey Bars and All Falls

	MONKEY BARS			ALL FALLS		
	N (151)	%	95% CI	N (3184)	%	95% CI
PRIMARY FINAL DIAGNOSIS						
Fracture	97	64.2	56.6 - 71.9	1189	37.3	35.6 - 39.0
Laceration/Abrasion	10	6.6	2.6 - 10.6	618	19.4	18.0 - 20.8
Soft Tissue Injury	23	15.2	9.5 - 20.9	641	20.1	18.7 - 21.5
Minor Head Injury/Concussion	13	8.6	4.1 - 13.1	502	15.8	14.5 - 17.1
Dislocation/Subluxation	1	0.7	0.0 - 2.0	58	1.8	1.3 - 2.3
Other^*^	7	4.6	1.3 - 7.9	176	5.5	4.7 - 6.3
ANATOMICAL LOCATION OF THE INJURY						
Head and Neck	26	17.2	11.2 - 23.2	1161	36.5	34.8 - 38.2
Upper Extremity	110	72.8	65.7 - 79.9	1291	40.5	38.8 - 42.2
Lower Extremity	6	4.0	0.9 - 7.1	553	17.4	16.1 - 18.7
Trunk	8	5.3	1.7 - 8.9	126	4.0	3.3 - 4.7
Abdomen/Pelvis	1	0.7	0.0 - 2.0	35	1.1	0.7 - 1.5
Unidentified	0	0.0	0.0 - 0.0	18	0.6	0.3 - 0.9

## Discussion

This study reports on the epidemiology of falls from monkey bars and other falls in children presenting to the emergency department in a Canadian city. Similar to Waltzman et al., we found a high proportion of fractures as the outcome of ED presentations for falls from monkey bars [11]. Our findings also concur with Mathison’s in that most of these fractures were in the upper extremities [6]. This highlights that ED clinicians must have a high degree of suspicion for arm fractures when assessing children who fell from monkey bars.

The reason for this heightened risk of fractures among school-aged children who fall from monkey bars is postulated to be secondary to a lack of physical maturity and dexterity. Younger children may not have the musculature and reflexes required to safely play on these climbing structures of significant height. Knowing this, the playground surface has been extensively studied in order to find the optimal material to reduce injuries secondary to falls. Multiple combinations have been reviewed from concrete to grass and rubber cushions. None of these seem to offer a unifying protective solution from injuries generated from monkey bars [8,13].

Between 1991 and 2005, the incidence of pediatric injuries at American playgrounds has been decreasing, except for the number of injuries from monkey bars [7]. This suggests that there is an inherent risk in playing on monkey bars regardless of the improving preventive measures. Are monkey bars too high? Are the strides too wide? Should we eliminate them from public parks? Further investigation will be required to establish the actual risk generated by these climbing structures. Nevertheless, falling from monkey bars is a high-risk mechanism of injury and should prompt physicians to investigate children for fractures upon presentation to the ED or office setting. It has been documented that child play at predefined increasing heights was not associated with an increasing fracture frequency and severity. The overall positive health effects secondary to risky outdoor play (eg. neuromuscular development, balance, agility, strength, etc.) may justify why children should not avoid this type of activity [14-15]. Nevertheless, educating parents on preventative behavior and considering the age and physical maturity of children when choosing playground activities may help reduce the number of serious injuries.

As expected, despite the temperate weather in Vancouver, we noticed a clear seasonal variation towards the summer months. We can hypothesize that in most other Canadian cities, with harsher winter conditions, we would observe a similar variation and a potential statistically significant association.

Our overall rate of fractures (37.3%) was similar to recent data from the USA (45.0%) [1]. In both studies, falls from variable standing heights were the most prominent mechanism involved. However, there were important differences between these two sets of data. First, it should be noted that the data from the USA solely relied on electronic medical record (EMR) coding of the chief complaint and discharge diagnosis while we manually reviewed all clinical history from medical charts. Secondly, the American data included children zero to 12 years old while ours focused on children age two to 17 years old. When limiting our analysis to children two to 12 years of age, our total number of falls was still more than twice the number of falls reported in Pitone’s study (5.8% of all ED visits versus 2.6%). This raises the concern that EMR code-based reviews may result in fewer subjects being identified for inclusion and, in this particular case, it underestimates the burden of pediatric falls.

Limitations

The BCCH only uses a partial electronic medical record. Nurses and physicians handwrite most of the information on the charts. Some charts were incomplete, illegible, or unclear on the exact type of injury. The lack of standardization in documenting a fall may have led to misinterpretations when the chart review was being performed and is the main limitation of this chart review. Also, some patient demographic data, such as “ethnicity,” was so poorly documented that we could not include it in the statistical analysis. It has been reported in one American study that African American and Native American children had a higher proportion of monkey bar injuries compared to Caucasian children (46.8% vs 44.4 vs 33.7%) [7]. Our unique Canadian demographic might be an alternative explanation for our results.

In addition, the type of playground equipment was not consistently recorded. It is possible that some of the falls from playground equipment were in fact from monkey bars. Since our analysis can’t account for this, it may result in a conservative estimate in our assessment of the odds of fracture among children who fall from monkey bars as compared to other fall mechanisms.

Another limitation of our study is that our target population was limited to children with an injury severe enough to present to the ED. We can safely assume that a substantial number of children fall due to various causes, including monkey bars, but do not seek medical attention. Therefore, our results cannot be directly interpreted as monkey bars being proportionally significant and an independent source of pediatric fractures.

In addition, our study could not specifically identify the location of the monkey bars and the composition of the landing surface. Based on Laforest et al., the risk of having a fracture or head injury is higher at a residential playground than at a public playground (OR 1.69) and grass should not be considered a safe surface under play equipment (OR 1.74) [13].

Finally, our study population was limited to one hospital in British Columbia, which, therefore, limits the generalizability of our results. Other Canadian cities are significantly more affected by seasonal variations, and the variation in ethnic diversity across Canada could influence the frequency of fractures secondary to monkey bars.

## Conclusions

Our study findings suggest that when evaluating a child who has had a fall, physicians should have a higher level of suspicion for a diagnosis of fracture if the child reportedly fell from monkey bars compared to falls from other mechanisms. Monkey bars represent a unique and significant etiology for pediatric injuries in early school-age children. It is warranted to educate parents and educators on the risks associated with playing on these climbing structures, and design and evaluate preventive measures targeting playground and sports-related falls.
